# Investigation of the Genotoxic Potential of the Marine Toxin C17-SAMT Using the In Vivo Comet and Micronucleus Assays

**DOI:** 10.3390/md20100619

**Published:** 2022-09-30

**Authors:** Zeineb Marzougui, Sylvie Huet, Anne-Louise Blier, Ludovic Le Hégarat, Haïfa Tounsi-Kettiti, Riadh Kharrat, Riadh Marrouchi, Valérie Fessard

**Affiliations:** 1 Laboratoire des Venins et Biomolécules Thérapeutiques, Institut Pasteur de Tunis, Université Tunis El Manar, 13 Place Pasteur, B.P. 74, Tunis-Belvédère, Tunis 1002, Tunisia; 2 National Institute of Agronomy, University of Carthage, Tunis 1082, Tunisia; 3 Unité de Toxicologie des Contaminants, Agence Nationale de Sécurité Sanitaire (ANSES), 10 B rue Claude Bourgelat, 35306 Fougères, France; 4 Laboratoire d’Anatomie Pathologique Humaine et Expérimentale, Institut Pasteur de Tunis, Université Tunis El Manar, 13 Place Pasteur, B.P. 74, Tunis-Belvédère, Tunis 1002, Tunisia

**Keywords:** marine biotoxins, C17-SAMT, genotoxicity, comet assay, micronucleus assay

## Abstract

The contaminant responsible for the atypical toxicity reported in mussels from Bizerte Lagoon (Northern Tunisia) during the last decade has been characterized as C17-sphinganine analog mycotoxin (C17-SAMT). This neurotoxin showed common mouse toxic symptoms, including flaccid paralysis and severe dyspnea, followed by rapid death. For hazard assessment on human health, in this work we aimed to evaluate the in vivo genotoxic effects of this marine biotoxin using the classical alkaline and modified Fpg comet assays performed to detect DNA breaks and alkali-labile sites as well as oxidized bases. The micronucleus assay was used on bone marrow to detect chromosome and genome damage. C17-SAMT induces a statistically insignificant increase in DNA tail intensity at all doses in the duodenum, and in the spleen contrary to the liver, the percentage of tail DNA increased significantly at the mid dose of 300 µg/kg b.w/d. C17-SAMT did not affect the number of micronuclei in the bone marrow. Microscopic observations of the liver showed an increase in the number of mitosis and hepatocytes’ cytoplasm clarification. At this level of study, we confirm that C17-SAMT induced DNA damage in the liver but there was no evidence of effects causing DNA oxidation or chromosome and genome damage.

## 1. Introduction

Food contamination can have different origins, including natural compounds produced by various kinds of organisms. Among the natural contaminants, mycotoxins are one of the most prevalent, leading to acute intoxications as well as long-term effects in humans [1]. Efforts to prevent mycotoxin contaminations remain not efficient enough, as fumonisin B1 has been found in nearly 50% of maize and maize-derived food in Europe, Canada, and Japan [2], and a global incidence of 60% for deoxynvalenol (DON) and 46% for zearalenon (ZEN) in unprocessed food-grade cereals has been documented between 2006 and 2016 [2]. Therefore, the worldwide population can be exposed to contaminated food, although at different levels depending on the country, the type of food, and the toxins involved. Nevertheless, the exposure risk is continuously growing due to environmental stress related to climate changes that provides optimal conditions for fungal contaminations [3]. Mycotoxins can be considered a chemical hazard but due to their biological origin, controlling exposure is more challenging than for anthropogenic chemicals [4]. Mycotoxins are divided into four groups according to the affected organ or tissue including hepatotoxins, immunotoxins, nephrotoxins, and neurotoxins [5]. Although humans can be exposed to a single type of mycotoxin, in a large number of cases, exposure to a combination of mycotoxins cannot be excluded. In fact, co-occurrence of mycotoxins can take place via ingestion of contaminated food, inhalation of spores, or skin contact from an environmental reservoir [6,7,8]. 

Although mycotoxin contamination is more frequent in crops and in the terrestrial environment, seafood can also be a source of mycotoxin dietary exposure. In fact, since its early days, the marine environment has been colonized by a large panel of microorganisms, including mycotoxin-producing fungi [9,10]. Besides their potential effects on fish and aquaculture products [11], marine mycotoxins are a potential hazard to human health due to their capability to bioaccumulate in different tissues and organs of seafood products [12]. With their high filtering capacities, bivalves are well-known to be the most contaminated seafood. However, knowledge of the contamination of shellfish and aquaculture livestock in general with mycotoxins remains limited [13]. The hypothesis of shellfish contamination with mycotoxins was first established to explain unknown toxic episodes along the French coasts in the early 1990s [14]. Further analysis proved the presence of *Aspergillus*, *Penicillium*, *Trichoderma*, and *Cladosporium* in the shellfish, sediment, and seawater from farming areas along the French coast [15]. Ever since then, other events of shellfish contamination with mycotoxins or with potential pathogenic fungi have been reported. In Brazil, cultured brown mussels *Perna perna* were found contaminated with *Pestalotiopsi* sp. [16], as well as farmed and wild mussels *Mytulis galloprovincialis* in the Adriatic Sea [17]. In the Sea of Japan (Russia), the contamination of yesso scallop *Mizuhopecten yessoensis*, Pacific oyster *Crassostrea gigas*, and bay mussel *M*. *trossulus* with a total of 52 species of potentially pathogenic filamentous fungi that were isolated from shells and internal organs, has been reported [18]. 

In the Mediterranean Sea, shellfish contamination has been correlated with major events of harmful algal blooms. Like all Mediterranean shellfish-farming countries, Tunisia has faced toxicity events devastating economic and social business. Indeed, the contamination of bivalve mollusks and the massive death of marine animals in Tunisia began during the 1990s in the Boughrara lagoon. In addition, researchers noticed a lower growth and a higher mortality of bivalves in the Bizerte lagoon. Analyses carried out on samples from the affected areas have proven the absence of toxigenic bacteria, toxic phytoplankton, and phycotoxins. Hence, toxic episodes in farmed mussels *M*. *galloprovincialis* were associated with the presence of marine microfungi (*Fusarium* sp., *Aspergillus* sp., and *Trichoderma* sp.) [19]. The severe toxicity of the mussel extracts was characterized by death within few minutes in the mouse bioassay. A bioassay-guided chromatographic separation followed by mass spectrometry detection was used to characterize the compound(s) responsible for toxicity and confirmed the implication of a 17-carbon short chain analogous to the sphinganine, named C17-Sphinganine Analog MycoToxin (C17-SAMT) with a molecular mass of 287.289 Da [19]. Electrophysiological investigations of the mouse neuromuscular system showed that C17-SAMT inhibits skeletal muscle contraction, which might explain some of the symptoms described during acute toxicity trials. This toxin has an LD_50_ in mice of 150 µg/kg, 750 µg/kg, and 900 µg/kg following intracerebroventricular, intraperitoneal, and oral administration, respectively [19]. 

In this study, we aimed to evaluate the in vivo genotoxic effects of this marine toxin for hazard assessment on human health. To do so, we performed an in vivo study in mice coupling two in vivo genotoxicity OECD test guidelines for comet assay (n°489) and micronucleus (n°474) after oral administration. The classical alkaline and modified Fpg comet assays were performed to detect DNA breaks and alkali-labile sites as well as oxidized bases on a panel of organs and tissues. The micronucleus assay was performed on bone marrow to detect chromosome and genome damage.

## 2. Results

### 2.1. Weight Changes 

**Figure 1:**
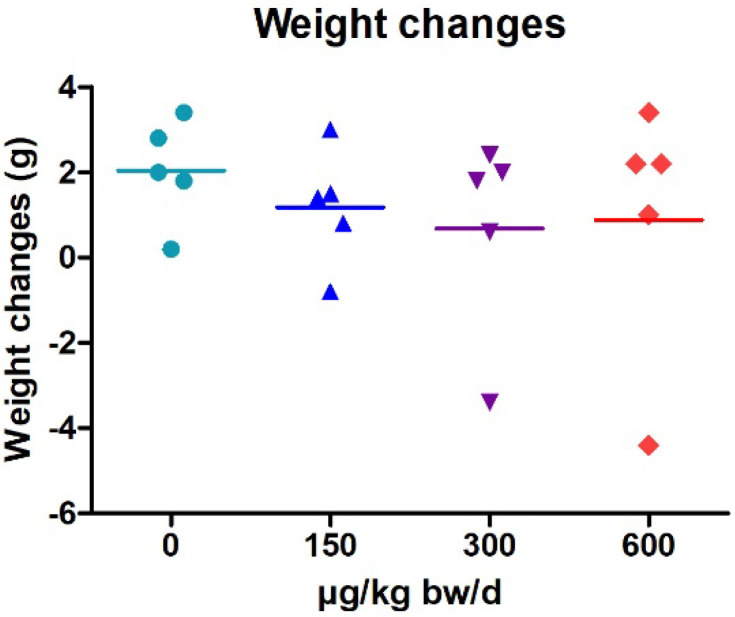
Individual results (five animals/group) of weight changes following three oral administrations of 150, 300, and 600 µg/kg of C17-SAMT. Lines indicate the mean of body weight change for each group.

Throughout the treatment period, the mice’s weight was recorded before each oral administration, and doses were adjusted. As shown in Figure 1, mean weight changes in mice dosed with C17-SAMT at 150, 300, and 600 μg/kg were not significantly different from the negative control group. However, at least one mouse per group lost weight, as shown in Figure 1. Two out of the three surviving mice treated with MMS (80 mg/kg) lost 4 g and 0.2 g at end of the treatment period.

### 2.2. Comet Assay

**Figure 2:**
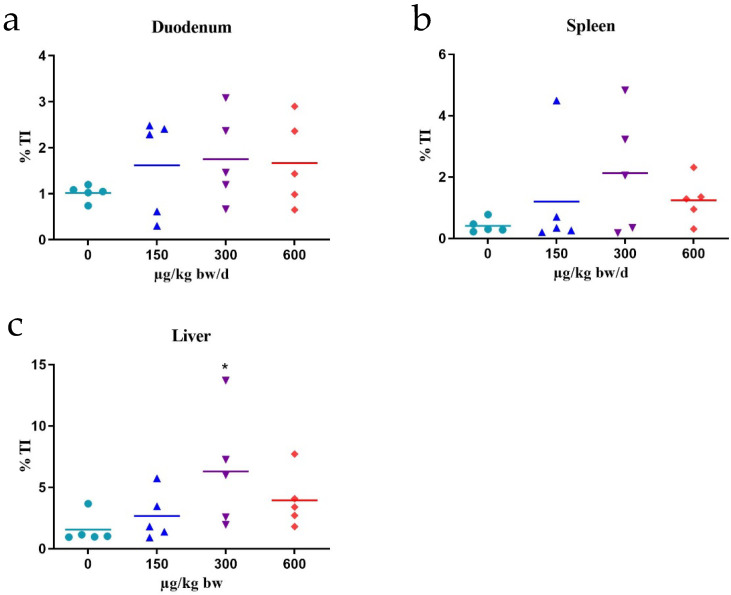
Individual results (five animals/group) obtained in the comet assay in the duodenum (**a**), spleen (**b**), and liver (**c**) after 3-day oral administration of different doses of C17-SAMT. DNA damage is expressed as median % of tail DNA intensity (%TI). Lines indicate mean of medians of %TI for each group. * *p* < 0.05. The positive control MMS induced 15.66 ± 6.48 and 22.73 ± 6.44%TI in the spleen and duodenum, respectively. In liver, 100% of hedgehogs was recorded.

C17-SAMT induced an increase in DNA tail intensity at all doses in the spleen and duodenum compared to the negative control, although not statistically significant (Figure 2). In the liver, a statistically significant increase in the percentage of tail DNA was observed at the mid dose of 300 µg/kg b.w, whereas for the low and the high dose, the %TI was increased but not statistically significant due to value dispersion (Figure 2).

**Figure 3:**
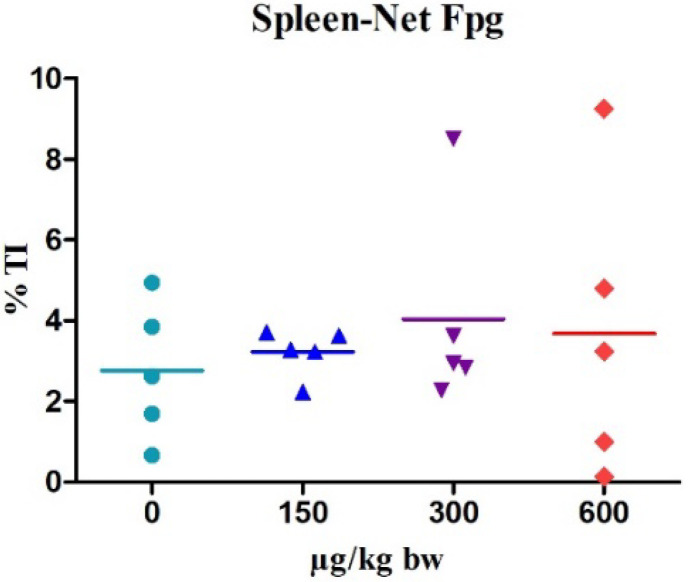
Individual results (five animals/group) obtained in the modified-comet assay in the spleen after 3-day oral administration of different doses of C17-SAMT. DNA damage is expressed as % Net-Fpg tail DNA intensity. Lines indicate the mean of medians of % TI for each group. The positive control MMS induced 100% of hedgehogs.

**Figure 4:**
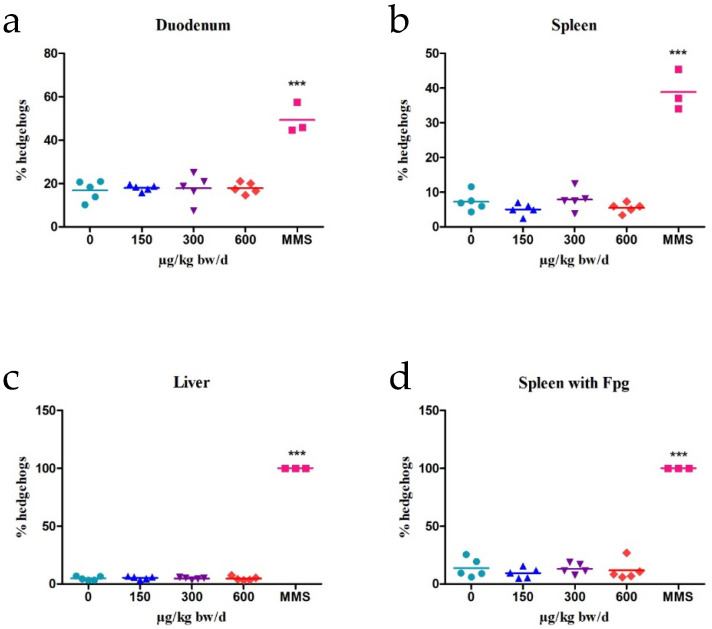
Individual results of hedgehogs’ frequency (five animals/group) obtained with the comet assay in different tissues and with the Fpg-modified comet assay in the spleen after 3-day oral administration of different doses of C17-SAMT (0—saline solution, 150, 300, and 600 µg/kg b.w.). MMS treatment was used for the positive control group. Lines indicate mean of % hedgehogs per group. (**a**): duodenum, (**b**): spleen, (**c**): liver, and (**d**): spleen with Fpg. *** *p* < 0.001.

In the modified-comet assay, we did not detect any significant increase in oxidative DNA damage in the spleen of mice exposed to C17-SAMT compared to the negative control (Figure 3). By contrast, MMS induced 100% of hedgehogs with Fpg in the spleen. The number of hedgehogs was low for all C17-SAMT-treated groups and in all organs collected compared to the negative control group (Table 1, Figure 4). By contrast, a statistically significant increase in the number of hedgehogs was observed for the MMS-treated group in all organs and tissues collected. 

### 2.3. Bone Marrow Micronucleus Test (BMMN)

The C17-SAMT toxin did not increase the number of MN-PCEs/1000 PCEs compared to the negative control group, regardless of the dose tested. The frequency of PCEs was not significantly different from the control group (Table 2). 

A significant increase in the percentage of MN-PCEs compared to the negative controls was obtained in the positive control group (MMS treatment).

### 2.4. Histopathological Observations

**Figure 5:**
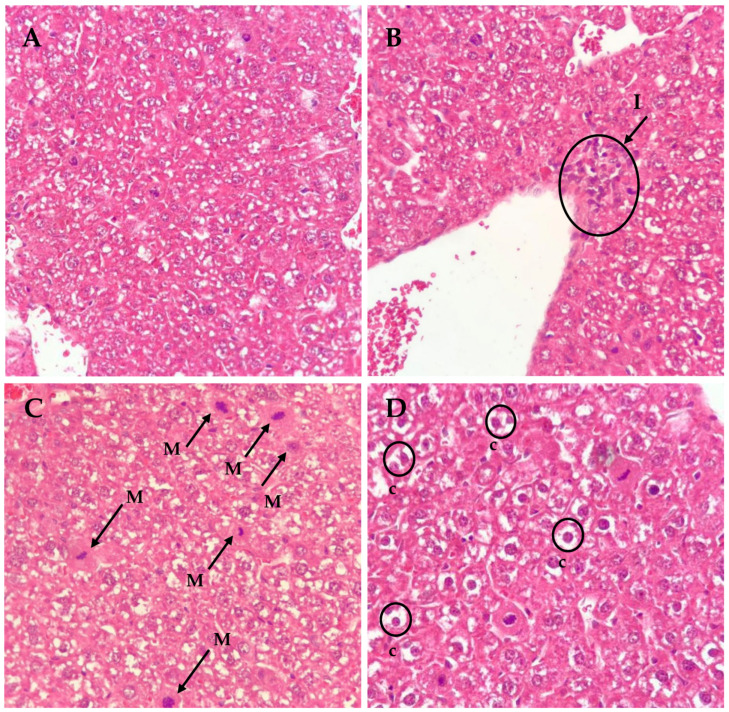
Examples of histopathological abnormalities observed in the liver from mice treated with three oral doses of C17-SAMT at 150 µg/kg b.w (**B**–**D**) compared to the negative control group (**A**). I: inflammatory infiltrate; M: mitosis; c: cytoplasm clarification. (Magnification ×40).

In order to discriminate if the positive result of the comet assay detected in the liver was due to true genotoxicity or to a necrotic or apoptotic effect of C17-SAMT, liver sections were analyzed for histopathological modifications. Compared to the negative control group, the livers of treated mice showed an increase in the number of mitosis, a clarified cytoplasm, and the presence of non-individualized vacuoles in the cytoplasm (Figure 5). In mice treated with 150 µg/kg b.w C17-SAMT, a slight inflammatory infiltration in the centrilobular vein was observed, an increased number of mitosis (mean of 65 mitosis/10 fields was estimated compared to less than 2 in negative control), and a clarified cytoplasm were recorded. However, no apoptosis or necrosis was observed, irrespective of the toxin dose. 

In mice treated with 300 and 600 µg/kg b.w., the same observations were recorded. In addition, at 600 µg/kg b.w., the presence of fragmented nuclei, without altered morphology, and atypical mitosis was outlined. The observations are summarized in Table 3.

## 3. Discussion

To our knowledge, this is the first study to evaluate the in vivo genotoxicity of the marine C17-sphinganine analog mycotoxin in mice following oral exposure using the OCDE guidelines n°489 and n°474 [20,21].

Using the bone marrow micronucleus assay, C17-SAMT failed to increase the frequency of micronucleated PCEs at all doses. However, as no decrease in the frequency of PCEs was obtained, we could not exclude that an insufficient amount of toxin reached the bone marrow, and therefore we could not reach a conclusion on the clastogenic/aneugenic effect of C17-SAMT.

In the alkaline and Fpg-modified comet assays, C17-SAMT did not induce any dose-dependent DNA damage in the spleen and duodenum collected 3 hours after three oral administrations of 150, 300, and 600 µg/kg (16.6%, 33.3% and 66.6% of LD50). However, an increase in DNA tail intensity was observed in liver cells at all doses with a statistical significance at the mid dose of 300 µg/kg b.w.. However, this does not comply with the criteria for a positive result described in the OECD guideline 489 (OECD, 2016 Ref guideline 489). In our study, only the first criterion (“*at least one of the test doses exhibits a statistically significant increase compared with the concurrent negative control”*) was met. The increase in tail DNA in the liver was not dose-related based on a trend test (*p* = 0.076). Moreover, we do not have sufficient historical control data for this species and the vehicle used. Therefore, the genotoxic effect of C17-SAMT observed in the liver should be further confirmed. Except for an increase in mitotic figures at the low and high doses, the histopathological analysis of liver sections did not show any cytotoxic effect, thus excluding a false-positive result induced by apoptosis or necrosis. 

When the ability of cells to eliminate and repair damage is compromised by excessive production of Reactive Oxygen Species ROS, macromolecules (DNA, lipids, and proteins) are oxidized [22]. However, the classical comet assay is generally not sensitive enough to detect DNA oxidative bases, and the addition of enzymes such as Fpg and OGG1 is required [23]. Nevertheless, in our study, the Fpg-modified comet assay in the spleen did not reveal any increase in DNA oxidative bases after oral exposure to C17-SAMT.

Other sphinganine-analog mycotoxins (SAMTs) have been reported to induce genotoxic effects in vivo in several publications [24,25,26,27,28,29]. However, positive results were either observed only at the highest dose [30], through a different route of administration (in vitro or in non-mammal species) [26,28], or after a longer period of treatment [30]. One of the most studied SAMTs, fumonisin B1 (FB1) induced DNA damage in rat spleen cells using the comet assay in a study conducted by Theumer and his collaborators [30]. Comets were scored on a scale from 0 (no tail) to 4–100% of DNA in the tail [31]. Up to 81.7% of treated mice presented a tail intensity ranging between 25% and 100%. A micronucleus increase of up to 7% in splenic cells from rats treated for 90 days with 100 mg/kg was recorded [30]. Moreover, FB1 is linked to esophageal cancer, liver cancer, and neural tube dysfunction [25], and it can induce various toxic effects, such as oxidative stress in primary rat hepatocytes [32], inhibition of mitochondrial respiration in rat primary astrocytes and human neuroblastoma cells (SH-SY5Y) [33], DNA damage in rats’ kidneys [34], and cellular cycle arrest in phase G2/M in rat C6 glioma cells [35]. It has been classified by IARC as potentially carcinogenic to humans in the group 2B [36]. 

*Alternaria* toxins, notably alternariol AOH, another sphinganine analog produced by *Alternaria* species, were tested on mice for toxicokinetics and genotoxicity evaluation. Results showed that AOH is not able to induce micronuclei formation in the bone marrow nor DNA damage in liver tissue of NMRI mice at high doses reaching 2000 mg/kg, which is probably due to the limited amount of toxin reaching the systemic circulation [37]. 

The family of sphinganine analogs is known to disturb the novo synthesis pathways of sphingolipids via the inhibition of the ceramide synthase [38]. Alteration in sphingolipid metabolism has been correlated with liver and kidney toxicity in rodents and farm animals, including carcinogenesis of liver and kidney in rodents [39,40,41,42,43,44,45]. As an analog to sphinganine, the hepatotoxicity observed with C17-SAMT might be related to the inhibition of ceramide synthase leading to the increase in intracellular sphinganine [38]. The accumulated sphinganine is metabolized into sphinganine 1-phosphate (Sa1P), which is subsequently cleaved into a fatty aldehyde and ethanolamine phosphate [40]. Therefore, sphinganine is considered cytotoxic [46,47,48]. 

As only one tested dose was statistically significant in liver using the comet assay, the genotoxic investigation of C17-SAMT was inconclusive. However, microscopic observations of liver sections showed an increase in the number of mitosis and cytoplasm clarification of hepatocytes, indicating a possible regeneration process activation. Additional studies need to be conducted to measure organ exposure after three oral administrations, and to reach a conclusion on the genotoxicity of C17-SAMT and the respective role of C17-SAMT and its metabolites.

## 4. Materials and Methods

### 4.1. Shellfish Sampling

Samples of mussels (*Mytilus galloprovincialis*) were collected monthly from Bizerte lagoon (Northern Tunisia). Sampling was carried out from shellfish farming areas and controlled by the ‘Commissariat Régional au Développement Agricole de Bizerte’ (CRDA, Bizerte). Samples were kept at 4 °C until analyzed. 

### 4.2. Chemicals

The C17-SAMT was extracted from contaminated mussels *M*. *galloprovincialis* as described previously by Marrouchi et al. (2013) [19], with a purification process using an HPLC bio-guided approach (Figure S1). The toxin concentration was estimated using an Agilent 1100 series analyzer with a Hypersil ODS-2 column (C18, 4.6 µm × 250 mm, 5 µm, ThermoScientific, Illkirch, France). To calibrate, a certified C17-SPA (10 mg/mL) solution was employed, and peak areas were measured to determine peak intensities. D-erythro-sphinganine (C17-SPA) from Avanti Polar Lipids (Alabaster, AL, USA). All other chemicals used were of the highest grade commercially available.

### 4.3. Animal Experimentation

Swiss male mice of 30 g (6–8 weeks) (Janvier Labs, Le Genest Saint-Isle, France), were housed in conventional cages and had free access to water and food. The temperature was monitored at 23 ± 1 °C and the light was pre-programmed on a light/dark cycle of 12 h/12 h. 

### 4.4. Selection of Dose Levels and Treatment

In accordance with the OECD guidelines n°489 and n°474, the Maximum Tolerated dose (MTD), defined “as the dose inducing slight toxic effects relative to the duration of the study period (for example, clear clinical signs such as abnormal behavior or reactions, minor body weight depression or target tissue cytotoxicity), but not death or evidence of pain, suffering or distress necessitating euthanasia”, should be the highest dose to be tested [22]. In order to determine the MTD for the C17-SAMT, a preliminary dose-range finding study was performed. A triple gavage in 45 h of C17-SAMT at 200 and 400 µg/kg b.w. did not induce any effect in mice; however, at 800 µg/kg b.w., C17-SAMT induced a severe effect in one out of three mice, with a bodyweight decrease of 20% 24 h after the second oral administration. Therefore, 600 mg/kg b.w. was considered as the MTD and the mid and low dose were set at ½ and ¼ of MTD, 300 and 150 mg/kg b.w. 

Male mice were divided into five groups (five mice/group). Three groups were exposed to C17-SAMT at 150, 300, and 600 mg/kg b.w. The negative control group received a solution of physiological serum. The positive control group was dosed with MMS at 80 mg/kg b.w. The gavage schedule followed a daily administration at 0, 24, and 45 h (10 mL/kg). Body weights were recorded before each administration and clinical signs were monitored daily.

This study was submitted to The French Ministry of Higher Education, Research, and Innovation, and received a favorable opinion from the ethics committee to which the establishment belongs (Opinion N°: n°2021-02-02-02), authorization APAFIS#28967-2021011211223011 v4.

### 4.5. Standard Comet Assay Protocol

Animals were sacrificed between 2 and 6 h after the last administration with an intraperitoneal sublethal injection of pentobarbital (60 g b.w.). Blood was collected from the vena cava and stored at 4 °C.

The duodenum was withdrawn, cut longitudinally, and cleaned with Hanks’ Balanced Salt Solution (HBSS) medium/10% DMSO (Sigma-Aldrich, St. Quentin-Fallavier, France). Cells were collected by scrapping the duodenum with a coverslip and filtered twice through a 150 µm filter. Liver cells were separated mechanically from small pieces with a Medimachine (BD Biosciences, Le-Pont-de-Claix, France). Spleen cells collected by aspiration. All samples were kept on ice until the preparation of slides. 

Comet assay was carried out as previously described by Tarantini et al. (2015) [49]. Briefly, isolated cells were centrifuged for 5 min at 136× *g*. Cells were mixed with 0.8% low melting-point (LMP) agarose and 65 μL of cell suspension was seeded on slides pre-coated with 1% normal melting agarose. Two slides/organ were prepared, except for the spleen (6 slides). After cell lysis (2.5 M NaCl, 0.1 M EDTA, 10 mM Tris-HCl pH 10, extemporarily added with DMSO 10% and 1% Triton X-100, (Sigma-Aldrich, St. Quentin-Fallavier, France)) for 1 h at 4 °C, DNA was allowed to unwind 20 min in electrophoresis buffer (0.3 M NaOH, 1 mM EDTA, pH13 (Sigma-Aldrich, St. Quentin-Fallavier, France)) before electrophoretic migration (24 min,0.7 V/cm, 300 mA). Slides were bathed two times for 5 min in a neutralizing solution (0.4 M Tris-HCl, pH 7.5), then fixed with ethanol 95% for 5 min.

### 4.6. Fpg-Modified Comet Assay Protocol

The bacterial formamidopyrimidine DNA glycosylase (Fpg) recognizes 8-oxo-7,8-dihydro-2′-deoxyguanine (8-oxodG) and alkylating damage in DNA, particularly ring-opened N7 guanine adducts (N-7 alkylguanines) [50] and was used to detect oxidative DNA damage only in spleen cells. After lysis incubation, the slides were washed in Fpg buffer (40 mM HEPES, 0.1 M KCl, 0.5 mM EDTA, pH 8.0 (Sigma-Aldrich, St. Quentin-Fallavier, France)) prior to a 30 min incubation with or without Fpg (3.6 units/slide) (Sigma-Aldrich, St. Quentin-Fallavier, France) at 37 °C. After two washes with PBS, the following steps (unwinding, electrophoresis, and neutralization) were identical to the standard comet assay protocol.

Coded slides were stained with propidium iodide (2 µg/mL in PBS) and observed with a fluorescence microscope (Leica DMR, Nanterre, France) equipped with a CCD-200E camera for scoring. Using the Comet Assay IV software (Perceptive Instruments, Haverhill, UK), 150 nucleoids were scored for each slide and 2 slides per organ and per animal. The percentage of DNA intensity in the tail (% Tail DNA) was chosen to evaluate DNA damage. The frequency of hedgehogs was also determined for each slide by manual scoring.

### 4.7. Bone Marrow Micronucleus Assay (BMMN)

The BMMN assay was performed according to the OECD guideline N°474 [21]. Briefly, the two femurs were flushed out with fetal bovine serum. After foaming, cells were kept at 4 °C.

Cells were centrifuged for 5 min at 210 g, spread on a microscope slide and allowed to air dry half a day before fixation in ethanol 96°. Two smears per animal were prepared. Slides were stained separately with pure and diluted May–Grünwald reagent and with 14% Giemsa (Fisher, IllkirchGraffenstaden, France). 

Micronuclei were scored by two independent scorers. Slides were examined under a bright field microscope and at least 2000 polychromatic erythrocytes (PCEs) per slide were scored. The frequency of micronucleated polychromatic erythrocytes (MN-PCEs) was determined for each slide. The ratio of PCEs to normochromatic erythrocytes (NCEs) was calculated to examine myelotoxicity.

### 4.8. Histopathological Observations

After mice euthanasia, the spleen, liver, and duodenum were rapidly removed and maintained in a fixative solution (formol 10%). The organs with positive results in the comet assay were further processed. Organ(s) were cut into slices, dehydrated in graded alcohol for 24 h, and then immersed in paraffin to form paraffined blocks. Thinner sections (3 to 5 mm thick) were subsequently cut using a Leica Ultracut microtome (Leica Microsystems, GmbH, Wetzlar, Germany) and stained with hematoxylin-eosin and iron hematoxylin. Microscopic examinations were carried out using a Zeiss light microscope. 

Microscopic observations focused on the presence of inflammation, cytoplasm clarification, necrosis, apoptosis, and mitosis. Inflammation and cytoplasm clarification were scored on a scale from 0 (absence), 1 (minimal), 2 (slight), 3 (moderate), 4 (marked) to 5-severe [51]. The number of mitosis per 10 microscopic fields was counted. 

### 4.9. Statistical Analysis

For the in vivo comet assay, the results were analyzed with a one-way ANOVA. For the BMMN assay, results were expressed as mean ± SD and analyzed with Pearson’s chi-square test with Yate’s correction.

## 5. Conclusions

In conclusion, our study reveals that C17-SAMT did not increase micronuclei in bone marrow but increased DNA damage in liver. As a clear positive result could not be obtained, the genotoxicity of C17-SAMT, including its mode of action, should be further investigated by in vitro testing.

## Figures and Tables

**Table 1:** Alkaline comet assay in different organs and Fpg modified comet assay in the spleen of male mice after 3-day oral administration of different doses of C17-SAMT (0—Phy. ser., 150, 300, and 600 µg/kg b.w.) or with MMS as the positive control. For each experimental group, the mean of % hedgehogs is reported. *** *p* < 0.001.

Organ	Treatment	Dose (µg/kg b.w)	% Hedgehogs
Duodenum	Control	0	16.78 ± 4.67
	C17-SAMT	150	17.94 ± 1.44
		300	17.71 ± 6.53
		600	17.86 ± 2.64
	MMS	80,000	49.3 ± 7.11 ***
Spleen	Control	0	7.23 ± 3.88
	C17-SAMT	150	5.04 ± 2.15
		300	7.85 ± 4.46
		600	5.5 ± 2.48
	MMS	80,000	38.84 ± 7.41 ***
Liver	Control	0	4.92 ± 1.98
	C17-SAMT	150	5.24 ± 2.46
		300	4.77 ± 1.47
		600	4.7 ± 2.23
	MMS	80,000	100 ***
Spleen Fpg+	Control	0	13.89 ± 9.66
	C17-SAMT	150	9.44 ± 4.81
		300	13.22 ± 5.92
		600	11.72 ± 8.92
	MMS	80,000	100 ***

**Table 2:** BMMN assay in mice following oral exposure to 150, 300, and 600 µg/kg of C17-SAMT. *** *p* < 0.001.

		MNPCEs/1000 PCEs	% PCEs
Doses (µg/kg b.w)		Mean ± SD	Mean ± SD
Control	0	1.2 ± 0.6	33 ± 0.07
C17-SAMT	150	1 ± 0.9	32 ± 0.07
	300	0.8 ± 0.9	36 ± 0.02
	600	1.6 ± 1.3	31 ± 0.03
MMS	80,000	13 ± 5.8 ***	27 ± 0.04

**Table 3:** Incidence for each histopathological endpoint analyzed on the liver of treated mice after 3-day oral administration of different doses of C17-SAMT (0—physiological serum, 150, 300, and 600 µg/kg b.w./d).

Treatment	Dose (µg/kg b.w)	Inflammation ^a^	Clarification ^a^	Necrosis	Apoptosis	Mitosis ^b^
CTRL	0	0	0	0	0	0
		0	0	0	0	0
		0	1	0	0	2
		0	0	0	0	0
		0	1	0	0	2
MMS	80,000	0	0	0	0	0
		0	0	0	0	1
		0	0	0	0	1
C17-SAMT	150	0	3	0	0	5
		1	2	0	0	>20
		0	2	0	0	>15
		0	2	0	0	5
	300	ns	ns	ns	ns	ns
		0	0	0	0	0
		0	3	0	0	5
		0	1	0	0	2
		1	2	0	0	2
	600	0	0	0	0	1
		0	3	0	0	3
		0	3	0	0	4
		0	3	0	0	3

a: 0—absence, 1—minimal, 2—slight, 3—moderate, 4—marked 5—severe; b: number of mitosis per 10 fields; ns: non-significant.

## Supplementary Materials

The following supporting information can be downloaded at: https://www.mdpi.com/article/10.3390/md20100619/s1, Figure S1: chromatograms of: (A) the water-soluble extract, which possessed the entire toxic activity, (B) the purified toxic fraction, and (C,D) the C-17 SAMT and the certified standard D-erythro-sphinganine (C17-SPA) co-eluted.
