# Supplementary material for: Investigation of the Genotoxic Potential of the Marine Toxin C17-SAMT Using the In Vivo Comet and Micronucleus Assays
Source: Mar Drugs. 2022 Sep 30;20(10):619. doi: 10.3390/md20100619 (PMC9604968; doi:10.3390/md20100619)
Supplement: Supplementary file 1 [file marinedrugs-20-00619-s001.zip › marinedrugs-1933791-supplementary.pdf]

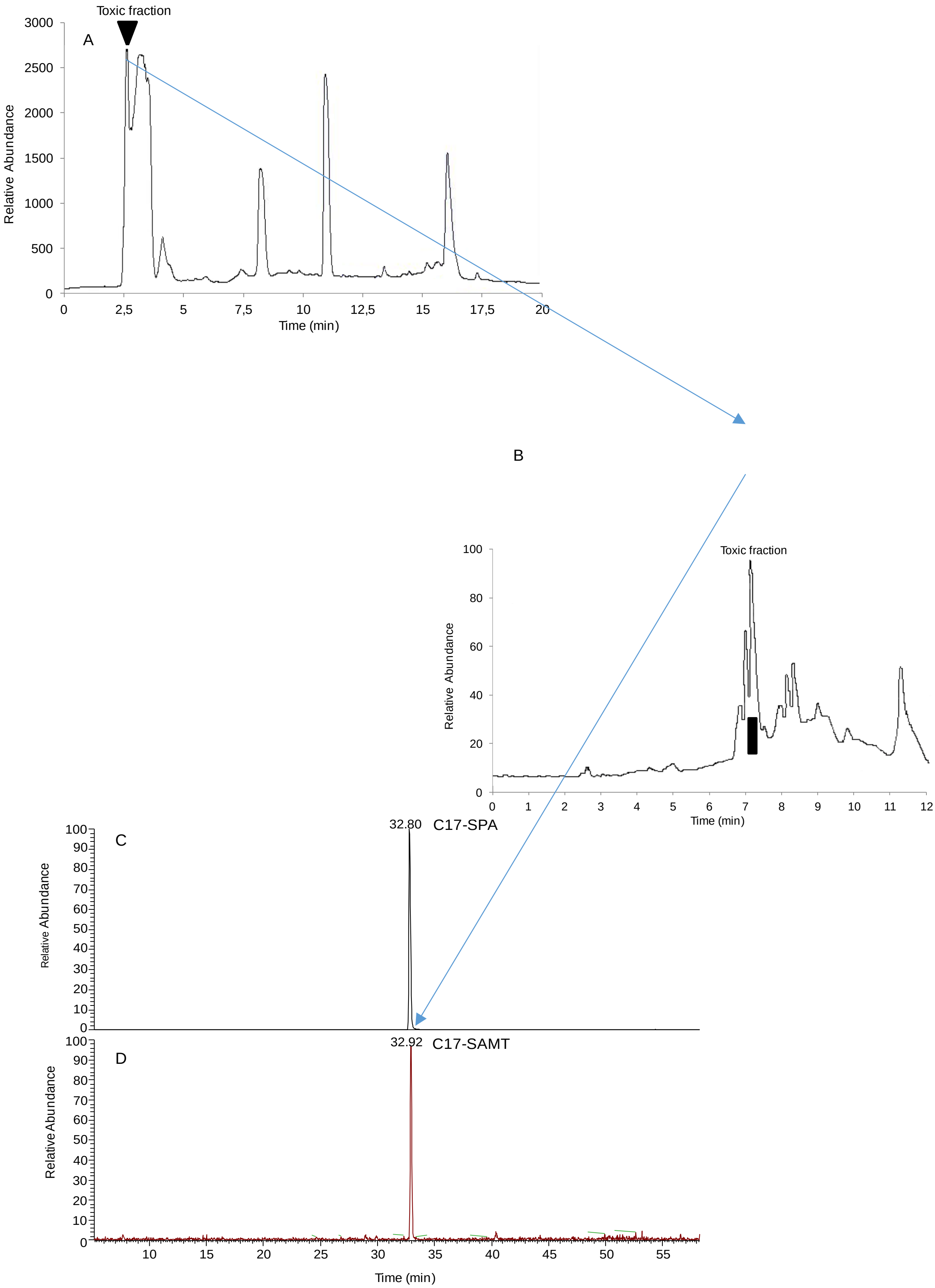

Figure S1: chromatograms of: (**A**) the water-soluble extract, which possessed the entire toxic activity, (**B**) the purified toxic fraction, and (**C**,**D**) the C-17 SAMT and the certified standard D-erythro-sphinganine (C17-SPA) co-eluted.
